# Examining the Mediating Role of Psychological Well‐Being in the Relationship Between Organizational Virtuousness and Proactive Work Behavior Among Nurses

**DOI:** 10.1155/jonm/4660713

**Published:** 2026-10-09

**Authors:** Ibrahim Abdullatif Ibrahim, Fahad M. Alhowaymel, Abdulaziz F. Abaoud, Doaa Mohamed Mohamed Ali Elzagh, Sabah Mohamed Ragab, Atallah Alenezi, Hala Gaber Elatroush, Mennat Allah G. Abou Zeid

**Affiliations:** ^1^ Department of Nursing Sciences College of Applied Medical Sciences Shaqra University Shaqra Saudi Arabia su.edu.sa; ^2^ Department of Nursing Administration Faculty of Nursing Mansoura University Mansoura Egypt mans.edu.eg; ^3^ Nursing Administration Department Faculty of Nursing Minia University Minia Egypt minia.edu.eg; ^4^ Nursing Administration Department Faculty of Nursing Beni Suef University Beni Suef Egypt bsu.edu.eg; ^5^ College of Nursing Imam Mohammad Ibn Saud Islamic University (IMSIU) Riyadh Saudi Arabia imamu.edu.sa; ^6^ Department of Nursing Administration and Education College of Nursing Prince Sattam Bin Abdulaziz University Al-Kharj Saudi Arabia psau.edu.sa; ^7^ Department of Nursing Administration Faculty of Nursing Ain Shams University Cairo Egypt asu.edu.eg

**Keywords:** nurses, organizational virtuousness, proactive work behavior, psychological well-being

## Abstract

**Background:**

Organizational virtuousness may be associated with proactive work behavior in healthcare organizations. However, the psychological processes underlying this association remain insufficiently understood.

**Aim:**

This study aimed to examine the indirect association between organizational virtuousness and proactive work behavior through psychological well‐being among nurses.

**Methods:**

This cross‐sectional study included a convenience sample of 206 nurses recruited from a renal and urology hospital in Minia Governorate, Egypt. Data were collected between April and June 2025 using self‐reported questionnaires comprising three validated scales and a demographic form. Structural equation modeling was used to test the hypothesized associations.

**Results:**

Organizational virtuousness was positively associated with nurses’ psychological well‐being (*B* = 0.91, *p* < 0.001) and proactive work behavior (*B* = 0.49, *p* < 0.001). Psychological well‐being was also positively associated with proactive work behavior (*B* = 0.21, *p* < 0.001). Furthermore, organizational virtuousness showed a significant indirect association with proactive work behavior through psychological well‐being (*B* = 0.19, *p* = 0.006; BC 95% CI [0.07, 0.37]).

**Conclusion:**

Organizational virtuousness was positively associated with nurses’ proactive work behavior both directly and indirectly through psychological well‐being. These findings suggest that a positive organizational climate characterized by virtuous practices may be relevant to nurses’ psychological well‐being and proactive workplace behavior.

**Implications for Nursing Management:**

Nursing leaders should consider fostering organizational environments characterized by virtuous practices, including supportive leadership, recognition, respectful communication, and policies that reinforce ethical conduct. Such practices may be associated with nurses’ psychological well‐being and proactive workplace behavior and may contribute to improved patient care and organizational effectiveness.

## 1. Introduction

Healthcare organizations worldwide are undergoing continuous transformation driven by technological innovation, digitalization, workforce shortages, increasing patient complexity, and evolving models of healthcare delivery. These changes require nurses not only to adapt to new clinical practices and organizational structures but also to proactively identify opportunities to improve patient care, enhance service quality, and contribute to organizational effectiveness [1, 2]. Accordingly, there is an increasing need for proactive approaches to maintaining high‐quality services, reducing waste, and enhancing patient safety and care [3]. Understanding the organizational conditions that encourage nurses to engage in proactive work behavior has therefore become an important priority for healthcare organizations seeking to improve quality and sustainability.

One organizational characteristic that may facilitate proactive employee behavior is organizational virtuousness, which refers to the collective demonstration of positive moral qualities, including trust, compassion, forgiveness, integrity, and optimism, embedded within organizational culture and interpersonal relationships [4, 5]. Organizations characterized by virtuous practices may foster respectful relationships, psychological safety, collaboration, and open communication, thereby creating an environment in which employees feel valued and supported [6].

Previous empirical studies have demonstrated that organizational virtuousness is positively associated with employee outcomes such as job satisfaction, organizational commitment, work engagement, innovative work behavior, and psychological well‐being [6–8]. Likewise, psychological well‐being has been identified as an important psychological factor associated with favorable employee outcomes in several occupational settings [8–10]. However, these studies have largely focused on outcomes such as engagement, performance, innovation, or commitment and have predominantly been conducted outside nursing contexts. Consequently, empirical evidence examining the role of psychological well‐being in the relationship between organizational virtuousness and proactive work behavior among nurses remains limited.

Although several studies have explored psychological well‐being as a mediating mechanism linking positive organizational characteristics with employee outcomes in non‐nursing and mixed occupational settings, considerably less empirical attention has been devoted to this mechanism in relation to proactive work behavior among nurses. Given the unique demands of nursing practice, including high workload, emotional labor, and rapidly changing clinical environments, findings from other occupational groups cannot be assumed to generalize directly to nursing settings.

Conceptually, organizational virtuousness and psychological well‐being represent distinct but related constructs. Organizational virtuousness reflects an organizational‐level characteristic embedded within workplace culture and interpersonal relationships through values such as trust, compassion, integrity, forgiveness, and optimism [4]. In contrast, psychological well‐being represents an individual‐level state of positive psychological functioning characterized by self‐acceptance, purpose, personal growth, and effective adaptation to workplace demands [11]. Thus, organizational virtuousness may provide contextual conditions that are associated with psychological well‐being, which may, in turn, be associated with proactive work behavior.

The present study is informed by three complementary theoretical perspectives. According to the Job Demands–Resources (JD–R) model, organizational virtuousness can be conceptualized as an organizational resource that may support employees’ motivation and psychological functioning [12]. Social Exchange Theory proposes that employees who perceive supportive, ethical, and caring organizational environments may reciprocate through discretionary behaviors that benefit the organization [13]. Furthermore, Affective Events Theory suggests that positive workplace experiences shape employees’ emotional and psychological functioning, which may subsequently be reflected in workplace behavior [14]. Integrating these perspectives provides a theoretically coherent explanation of how organizational virtuousness may be associated with proactive work behavior both directly and indirectly through psychological well‐being.

Building on previous research, the present study examines a theoretically informed model linking organizational virtuousness, psychological well‐being, and proactive work behavior within a nursing context. Specifically, the study investigates whether psychological well‐being is associated with the relationship between organizational virtuousness and proactive work behavior among nurses in an Egyptian tertiary hospital. By integrating the JD‐R model, Social Exchange Theory, and Affective Events Theory, this study provides context‐specific evidence that contributes to a better understanding of the organizational and psychological factors associated with proactive work behavior in nursing practice.

## 2. Literature Review and Hypotheses Development

### 2.1. Organizational Virtuousness and Proactive Work Behavior

Proactive work behavior refers to future‐focused, change‐oriented, and self‐initiated actions undertaken by employees [15]. This behavior reflects a bottom‐up approach in which employees actively shape and influence their work environment, emphasizing personal agency and responsibility. Previous research has shown that proactive work behavior is associated with important outcomes such as job performance, creativity, and service quality. In healthcare settings, such behavior is particularly relevant because it may contribute to continuous improvement in patient care and organizational effectiveness [16].

Proactive work behavior is typically conceptualized as multidimensional and includes taking charge, voice behavior, individual innovation, and proactive problem prevention. Taking charge involves voluntary efforts to bring about constructive changes in work practices. Voice behavior reflects the expression of constructive suggestions intended to improve organizational functioning. Individual innovation refers to the generation and implementation of novel ideas that support organizational goals [17]. Proactive problem prevention involves anticipating potential problems and taking steps to reduce their occurrence or recurrence [18, 19].

Organizational virtuousness refers to shared perceptions of positive organizational characteristics, such as compassion, integrity, trust, and forgiveness. Such an environment may provide conditions that encourage employees to engage in constructive and change‐oriented behaviors. These conditions may support open communication, psychological safety, and interpersonal trust, which are relevant antecedents of proactive behavior [10]. Employees in such contexts may also reciprocate positive organizational treatment through constructive contributions, consistent with social exchange processes.

Empirical studies have reported positive associations between organizational virtuousness and various organizational outcomes, including job satisfaction, organizational commitment, and positive work behavior [4, 5]. Grounded in the JD‐R model [12] and Social Exchange Theory [13], organizational virtuousness can be conceptualized as a contextual organizational resource that may be associated with favorable employee attitudes and behaviors. From the JD‐R perspective, supportive organizational resources may enhance employees’ motivation and engagement, which may facilitate proactive work behavior. From a Social Exchange Theory perspective, favorable and virtuous organizational conditions may encourage employees to reciprocate through positive discretionary behaviors. Accordingly, organizational virtuousness may provide both motivational and relational conditions that are associated with nurses’ proactive work behavior. Therefore, the following hypothesis was proposed:

 H1: Organizational virtuousness is positively associated with proactive work behavior among nurses.

### 2.2. Organizational Virtuousness and Psychological Well‐Being

Psychological well‐being refers to an individual’s optimal psychological functioning, including positive affect, self‐acceptance, purpose in life, personal growth, and effective stress management [10]. Within organizational research, Affective Events Theory provides a useful framework for understanding how workplace conditions are associated with employees’ emotional experiences and subsequent psychological states [14]. The Affective Events Theory suggests that workplace environments shape affective experiences, which accumulate over time and influence psychological well‐being.

From this perspective, organizational virtuousness may be viewed as a contextual condition that is associated with more frequent positive affective experiences. Work environments characterized by compassion, trust, integrity, and forgiveness may contribute to improved psychological functioning by providing emotional and relational support. Previous studies in non‐nursing contexts have reported positive associations between organizational virtuousness and employee well‐being [6, 20]. However, evidence in nursing populations remains limited despite the high emotional demands of clinical practice.

Accordingly, the following hypothesis is proposed:

 H2: Organizational virtuousness is positively associated with psychological well‐being among nurses.

### 2.3. Psychological Well‐Being and Proactive Work Behavior

Psychological well‐being reflects positive psychological functioning and the presence of positive emotional and cognitive resources [11]. Individuals with higher psychological well‐being are more likely to experience energy, resilience, motivation, and cognitive flexibility, which may enable them to engage in proactive behavior at work [21, 22]. Prior research suggests that employees with higher psychological well‐being are more likely to demonstrate adaptive and constructive work behavior, including proactive engagement in improving work processes and patient care [23, 24]. Therefore, psychological well‐being may be associated with proactive work behavior among nurses.

 H3: Psychological well‐being is positively associated with proactive work behavior among nurses.

### 2.4. Psychological Well‐Being as a Mediating Mechanism

The JD‐R model proposes that job and organizational resources are associated with employee outcomes through motivational and psychological processes [12]. Within this framework, organizational virtuousness may function as a contextual resource that is associated with employees’ psychological well‐being. In turn, psychological well‐being may be associated with proactive work behavior by enhancing motivation, energy, and cognitive capacity.

Similarly, Affective Events Theory suggests that workplace conditions influence emotional experiences, which subsequently shape psychological states and behavioral outcomes [14]. From this perspective, organizational virtuousness may be associated with psychological well‐being, which in turn may be associated with proactive work behavior.

Empirical studies provide indirect support for this pathway. Arshad et al. [20] found that organizational virtuousness is associated with employee well‐being in the postpandemic context, which in turn relates to in‐role and extra‐role performance behavior. Ho et al. [10] showed that virtuous organizational conditions are linked to psychological resources and well‐being over time, which subsequently relate to extra‐role performance and commitment. Although their focus was on psychological capital, their findings support the broader role of psychological mechanisms in linking virtuous contexts to work outcomes. In addition, Aubouin‐Bonnaventure et al. [25] reported that virtuous organizational practices are associated with employee well‐being indicators, with partial mediation via psychological resources.

Collectively, these studies suggest that psychological well‐being may serve as an explanatory mechanism in the relationship between organizational virtuousness and proactive work behavior. However, given the cross‐sectional design and differences in constructs across studies, this mediation should be interpreted as a theoretically informed association rather than evidence of causality. Therefore, the following hypothesis is proposed:

 H4: Organizational virtuousness is indirectly associated with proactive work behavior through psychological well‐being among nurses.

### 2.5. Aim of the Study

This research aimed to investigate the association between organizational virtuousness and proactive work behavior, focusing on the indirect role of psychological well‐being of nurses.

## 3. Methods

### 3.1. Study Design, Participants and Setting

This cross‐sectional study was conducted among bedside nurses working at the Renal and Urology Hospital in Minia Governorate, Egypt. The hospital is a specialized tertiary care facility providing renal, urological, dialysis, surgical, and related inpatient and outpatient services. The cross‐sectional design was considered appropriate for examining theoretically grounded associations among organizational virtuousness, psychological well‐being, and proactive work behavior at a single point in time. This design is particularly suitable for testing mediation models using structural equation modeling (SEM) in exploration and theory‐testing contexts where temporal sequencing is not yet established.

The target population comprised full‐time staff nurses providing direct patient care. According to hospital administrative records, the nursing workforce during the data collection period consisted of 451 staff nurses. A convenience sampling technique was used to recruit eligible participants. Nurses were included if they: (1) were full‐time staff nurses, (2) had at least 1 year of clinical experience, and (3) agreed voluntarily to participate in the study. Nurses in managerial or supervisory positions, those on internship or training programs, and those on leave during the data collection period were excluded to ensure homogeneity of clinical exposure and role responsibilities. This study followed the Strengthening the Reporting of Observational Studies in Epidemiology (STROBE) guidelines to ensure transparent and standardized reporting of observational research.

Sample size determination was conducted using Soper’s A‐priori sample size calculator for SEM [26]. The calculation considered a statistical power of 0.80, a significance level of 0.05, a medium anticipated effect size of 0.30 (*f* ^2^), based on prior empirical literature [27], 12 latent variables, and 36 observed indicators, reflecting the hierarchical measurement structure of the study constructs. Specifically, organizational virtuousness comprised five first‐order factors and one second‐order factor, while proactive work behavior comprised four first‐order factors and one second‐order factor; psychological well‐being was represented by a single latent factor. Based on these parameters, the calculator indicated the minimum required sample size was 200 participants. A total of 240 questionnaires were distributed, of which 206 were returned fully completed and included in the final analysis, yielding an 85.8% response rate. Thus, the final analyzable sample exceeded the prespecified minimum requirement. In addition, an RMSEA‐based power analysis was conducted using the approach proposed by MacCallum et al. [28]. With 206 participants, 582 degrees of freedom, a significance level of 0.05, an RMSEA of 0.05 under the null hypothesis, and an RMSEA of 0.08 under the alternative hypothesis, the analysis indicated approximately 100% statistical power to distinguish the specified levels of model fit. This provides additional support for the adequacy of the final sample for evaluating the overall fit of the covariance‐based SEM.

### 3.2. Instrumentation and the Study Variables

#### 3.2.1. Independent Variable: Organizational Virtuousness

Organizational virtuousness was measured using a validated 15‐item scale developed by Cameron et al. [4]. The scale conceptualizes organizational virtuousness as a multidimensional construct comprising five dimensions: optimism, trust, compassion, integrity, and forgiveness, with three items representing each dimension. Responses were rated on a five‐point Likert scale ranging from 1 (*strongly disagree*) to 5 (*strongly agree*), with higher scores indicating stronger perceptions of organizational virtuousness. Item scores were averaged to generate an overall organizational virtuousness score, with possible scores ranging from 1 to 5. Higher mean scores indicate stronger perceptions of organizational virtuousness. The original instrument has demonstrated a stable five‐factor structure with strong internal consistency reliability (*α* = 0.83–0.89) and excellent sampling adequacy (KMO = 0.954), supporting its validity in prior research.

#### 3.2.2. Mediating Variable: Psychological Well‐Being

We utilized the 8‐item Psychological Flourishing Scale to assess the psychological well‐being levels of nurses [29]. Items were rated on a seven‐point Likert scale ranging from 1 (*strongly disagree*) to 7 (*strongly agree*), with higher scores indicating greater psychological well‐being. Item scores were averaged to obtain an overall psychological well‐being score, with possible scores ranging from 1 to 7. Higher mean scores indicate greater psychological well‐being. The original instrument has demonstrated strong psychometric properties, including good internal consistency reliability (Cronbach’s α = 0.87) and established construct and convergent validity.

#### 3.2.3. Dependent Variable: Proactive Work Behavior

Proactive work behavior was measured using a 13‐item scale to assess nurses’ tendency to take initiative and engage in proactive behavior in various situations [30]. The scale includes four dimensions: problem prevention (3 items), individual innovation (4 items), voice (4 items), and taking charge (3 items). Responses were recorded on a five‐point Likert scale ranging from 1 (*very infrequent*) to 5 (*very frequently*), with higher scores indicating higher levels of proactive behavior. Item scores were averaged to calculate an overall proactive work behavior score, with possible scores ranging from 1 to 5. Higher mean scores indicate higher levels of proactive work behavior.

The original instrument has demonstrated a stable four‐factor structure, strong internal consistency reliability (*α* = 0.75–0.83), and evidence of convergent and discriminant validity.

#### 3.2.4. Descriptive Variables: Demographic Characteristics

A demographic form was used to collect data including age, gender, marital status, education, work shift, and years of nursing experience. These variables were collected solely for the purpose of describing the sample and characterizing the study population.

#### 3.2.5. Cross‐Cultural Adaptation Procedure

All instruments underwent cross‐cultural adaptation following the standardized guidelines proposed by Beaton et al. [31], ensuring semantic, idiomatic, experiential, and conceptual equivalence between the original English versions and the Arabic context. The adaptation process included forward translation, back‐translation, and synthesis, followed by expert panel review conducted by five experts in nursing research to ensure linguistic accuracy and content validity.

A pilot study was conducted with 21 nurses to evaluate the clarity, comprehensibility, and feasibility of the questionnaire and to estimate the time required for completion. Internal consistency reliability was assessed using Cronbach’s alpha, demonstrating acceptable reliability for all study instruments: organizational virtuousness (*α* = 0.82), psychological well‐being (*α* = 0.86), and proactive work behavior (*α* = 0.91). Because the pilot sample was not sufficiently large for comprehensive psychometric evaluation, construct validity, including convergent and discriminant validity, was assessed using the full study sample (*N* = 206). The pilot participants were excluded from the main study.

### 3.3. Data Collection

The researchers collected data over a 3‐month period, from April to June 2025. The support of head nurses from each ward was obtained to facilitate access to participants; however, head nurses were only involved in coordinating unit entry and were not engaged in questionnaire distribution or collection, and they did not have access to completed responses in order to minimize potential coercion or response bias.

Paper‐based questionnaires were distributed to staff nurses during their working hours within their respective units. Each questionnaire included a cover page explaining the study objectives and emphasizing voluntary participation, anonymity, and confidentiality. To further protect participants’ privacy, nurses were provided with sealed envelopes to return completed questionnaires, which were then deposited into securely locked collection boxes placed in the nursing managers’ offices in each unit. To ensure completeness of the data and minimize missing responses, all questionnaires were reviewed immediately upon return. Any questionnaire with missing or incomplete items was checked on the spot, when possible, by requesting participants to complete omitted responses, while maintaining voluntary participation and without any pressure to respond. Completion of the questionnaire required approximately 10–15 min.

### 3.4. Statistical Analysis

Statistical analysis was performed using IBM SPSS Statistics Version 27.0 and AMOS Version 24.0. Descriptive statistics (means, standard deviations, frequencies, and percentages) were used to summarize participants’ demographic characteristics and study variables. Pearson correlation analysis was conducted to examine the bivariate relationships among the study constructs.

The hypotheses were tested using the two‐step approach proposed by Anderson and Gerbing [32]. In the first step, confirmatory factor analysis (CFA) was conducted to assess the measurement model in terms of reliability, convergent validity, and discriminant validity. In the second step, SEM was employed to test the hypothesized direct and indirect relationships among the study variables. A bootstrapping procedure with 5000 resamples was applied to examine the significance of mediation effects. Prior to SEM analysis, data screening procedures were conducted. Normality was assessed using skewness and kurtosis values, which were within acceptable thresholds (±2). No missing data were observed in the dataset, and multivariate outlier analysis using Mahalanobis distance indicated the absence of influential outliers.

Model fit was evaluated using multiple goodness‐of‐fit indices, including the chi‐square to degrees of freedom ratio (*χ* ^2^/df), Goodness of Fit Index (GFI), Incremental Fit Index (IFI), Tucker–Lewis Index (TLI), Comparative Fit Index (CFI), Root Mean Square Error of Approximation (RMSEA), and Standardized Root Mean Square Residual (SRMR). Acceptable model fit was determined by *χ* ^2^/df ≤ 3.0, GFI, IFI, TLI, and CFI ≥ 0.90, RMSEA ≤ 0.08, and SRMR ≤ 0.08, in accordance with established recommendations [33]. All statistical tests were two‐tailed, and a *p* value < 0.05 was considered statistically significant. Consistent with Becker et al. [34], demographic variables were not included as statistical controls, as their use without a clear theoretical rationale may bias parameter estimates. Given the study’s focus on the associations among organizational virtuousness, psychological well‐being, and proactive work behavior, demographic characteristics were reported descriptively to characterize the sample but were not entered into the hypothesis‐testing models.

### 3.5. Ethical Considerations

Ethical approval for the study was obtained from the Research Ethics Committee of the Faculty of Nursing, Minia University (Reference Number: REC202432). The study was conducted in accordance with the principles of the Declaration of Helsinki. Prior to participation, all nurses were provided with clear information regarding the study objectives, procedures, potential risks, and benefits. Written informed consent was obtained from all participants before data collection. Participation was entirely voluntary, and respondents were informed of their right to withdraw from the study at any time without penalty or consequences. Confidentiality and anonymity were strictly maintained throughout the research process, and all data were used exclusively for academic research purposes.

## 4. Results

### 4.1. Demographic Characteristics of the Participants

The study participants had a mean age of 23.66 ± 3.97 years, with 92.7% aged between 20 and 30 years. Females accounted for 55.8% of the sample, while males accounted for 44.2%. Regarding marital status, 76.2% of participants were unmarried. In terms of educational attainment, 66.0% held a bachelor’s degree, 26.2% held a technical degree, and 7.8% were pursuing postgraduate studies. Regarding work patterns, 86.9% were engaged in rotating shifts, 9.7% worked morning and evening shifts, and 3.4% worked morning shifts exclusively. Additionally, 80.1% of participants reported 1–5 years of work experience (Table 1).

**TABLE 1:** Characteristics of the participants (*N* = 206).

Characteristics	*N*	%
Age		
20–30	191	92.7
31–40	15	7.3
Mean ± SD	23.66 ± 3.97	
Gender		
Male	91	44.2
Female	115	55.8
Marital status		
Unmarried	157	76.2
Married	49	23.8
Education		
Technical degree	54	26.2
Bachelor degree	136	66.0
Postgraduates studies	16	7.8
Working shift type		
Morning shift	7	3.4
Morning and evening	20	9.7
Rotating shifts	179	86.9
Experience		
1–5	165	80.1
6–10	32	15.5
> 10	9	4.4

### 4.2. CFA‐Measurement Model

CFA was conducted to assess the measurement model based on the hypothesized latent constructs of organizational virtuousness, psychological well‐being, and proactive work behavior. The three‐factor model demonstrated an acceptable fit to the data (*χ* ^2^/df = 1.48, GFI = 0.91, CFI = 0.92, TLI = 0.91, IFI = 0.91, RMSEA = 0.06) and provided a better fit than the alternative models, including a two‐factor model in which organizational virtuousness and psychological well‐being were specified as a single factor (*χ* ^2^/df = 3.11, GFI = 0.62, CFI = 0.74, TLI = 0.73, IFI = 0.75, RMSEA = 0.10) and a one‐factor model in which all constructs were combined (*χ* ^2^/df = 4.29, GFI = 0.45, CFI = 0.60, TLI = 0.58, IFI = 0.61, RMSEA = 0.12). These results support the empirical distinctiveness of the latent constructs as specified in the measurement model (Table 2).

**TABLE 2:** Summary of CFA analysis (*N* = 206).

Models	*χ* ^2^	df	*χ* ^2^/df	GFI	IFI	TLI	CFI	RMSEA
One factor model: OV + PW + PWB	2552.16	594	4.29	0.45	0.61	0.58	0.60	0.12
Two‐factor model (OV and PW combined), PWB	1843.18	593	3.11	0.62	0.75	0.73	0.74	0.10
Hypothesized three‐factor model: OV, PW, PWB	1029.59	559	1.84	0.91	0.91	0.91	0.92	0.06

*Note:* PW: Psychological well‐being.

Abbreviations: OV, organizational virtuousness; PWB, proactive work behavior.

Regarding common method variance, Harman’s single‐factor test indicated that the first unrotated factor accounted for 40.17% of the total variance, which is below the commonly referenced threshold of 50% [35]. To assess multicollinearity, tolerance and variance inflation factor (VIF) values were examined. The tolerance values (0.711) exceeded the recommended threshold of 0.20, and the VIF values (1.407) were well below the recommended threshold of 5.0, indicating that multicollinearity was not a concern.

Convergent validity is confirmed when the factor loadings of scale items and the average variance extracted (AVE) exceed 0.5, and when composite reliability (CR) is greater than 0.7 [36]. The AVE values for the constructs in this study ranged from 0.508 to 0.530, while the factor loadings for all survey items fell between 0.540 and 0.823, exceeding the acceptable threshold of 0.5. The CR values varied between 0.891 and 0.940, all surpassing the 0.7 threshold, thus confirming convergent validity. Discriminant validity was assessed using the Fornell–Larcker criterion and the HTMT. The square roots of the AVE values exceeded the inter‐construct correlations, and all HTMT values were below the recommended threshold of 0.85 [37, 38], supporting discriminant validity. Reliability was evaluated using Cronbach’s alpha coefficients, all of which exceeded 0.70, indicating satisfactory internal consistency of the study measures [39] (Table 3).

**TABLE 3:** Descriptive statistics, reliability, validity, and correlation analysis (*N* = 206).

The study variables	Mean ± SD	Factor loading	*α*	CR	AVE	MSV	1	2	3
1. Organizational virtuousness	3.81 ± 0.67	0.633–0.823	0.91	0.940	0.513	0.395	**0.716**	*0.565*	*0.617*
2. Psychological well‐being	5.67 ± 0.93	0.597–0.834	0.93	0.936	0.530	0.394	0.538^∗∗∗^	**0.728**	*0.548*
3. Proactive work behavior	3.62 ± 0.68	0.540–0.778	0.88	0.891	0.508	0.317	0.589^∗∗∗^	0.506^∗∗∗^	**0.713**

*Note:* Bolded values on the diagonals represent the square root of AVE; off‐diagonal values are construct correlations; italicized values represent heterotrait–monotrait (HTMT) ratios.

Abbreviations: AVE, average variance extracted; CR, composite reliability; MSV, maximum shared variance; SD, standard deviation.

^∗∗∗^ *p* < 0.001.

### 4.3. Descriptive and Correlation Statistics Among the Study Variables

The mean score for organizational virtuousness was 3.81 (±0.67). The mean score for psychological well‐being was 5.67 (±0.93). The mean score for proactive work behavior was 3.62 (±0.68). Correlation analysis indicated a significant moderate positive association between organizational virtuousness and psychological well‐being (*r* = 0.538, *p* < 0.001). A significant moderate positive association was also found between organizational virtuousness and proactive work behavior (*r* = 0.589, *p* < 0.001). Additionally, a significant moderate positive association was also found between psychological well‐being and proactive work behavior (*r* = 0.506, *p* < 0.001) (Table 3).

### 4.4. Hypotheses Testing (Structural Model)

Organizational virtuousness was positively associated with psychological well‐being (*B* = 0.91, *p* < 0.001) and proactive work behavior (*B* = 0.49, *p* < 0.001). Psychological well‐being was also positively associated with proactive work behavior (*B* = 0.21, *p* < 0.001). The indirect association between organizational virtuousness and proactive work behavior via psychological well‐being was statistically significant (*B* = 0.19, *p* = 0.006), with a bias‐corrected 95% confidence interval of [0.07, 0.37], indicating evidence of partial mediation. The overall association between organizational virtuousness and proactive work behavior was significant (*B* = 0.68), with a bias‐corrected 95% confidence interval of [0.41, 1.10]. Model fit indices indicated an acceptable fit to the data (*χ* ^2^/df = 2.11, GFI = 0.91, CFI = 0.93, TLI = 0.92, IFI = 0.93, RMSEA = 0.07). The structural model explained 34% of the variance in psychological well‐being (*R* ^2^ = 0.34) and 45% of the variance in proactive work behavior (*R* ^2^ = 0.45), indicating that the model had moderate explanatory power, as shown in Table 4 and Figure 1.

**TABLE 4:** Maximum likelihood estimates of the mediation model (*N* = 206).

Path	*B*	SE	*t*	*p*	BC 95% CI	
					Lower bounds	Upper bounds
Organizational virtuousness to psychological well‐being	0.91	0.16	5.86	< 0.001	0.63	1.42
Psychological well‐being to Proactive work behavior	0.21	0.06	3.61	< 0.001	0.08	0.37
Organizational virtuousness to proactive work behavior	0.49	0.11	4.52	< 0.001	0.22	0.81
Indirect effect	0.19			0.006	0.07	0.37
Total effect	0.68			0.004	0.41	1.10

*Note:* Unstandardized coefficients are reported; bootstrap resamples = 5000. Squared multiple correlations (*R* ^2^): psychological well‐being = 0.34; Proactive work behavior = 0.45.

Abbreviations: BC, bias‐corrected; CI, confidence interval; SE, standard error.

**FIGURE 1:**
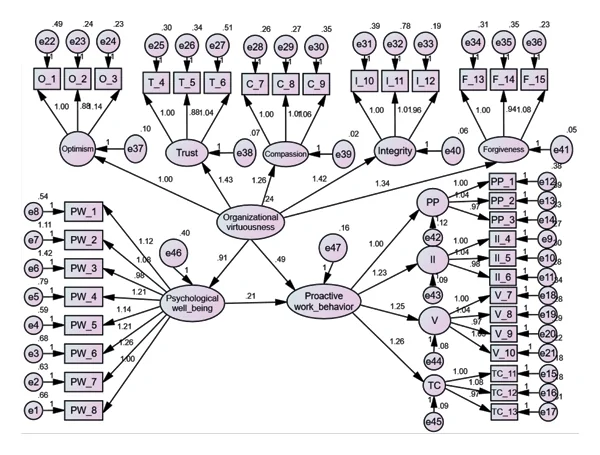
The mediation model (*N* = 206). Abbreviations: PP: problem prevention, II: individual innovation, V: voice, TC: taking charge.

## 5. Discussion

This study examined the relationships between organizational virtuousness, psychological well‐being, and proactive work behavior among nurses, with particular attention to the potential mediating role of psychological well‐being. The findings demonstrated significant positive associations among the study variables and suggest that psychologically healthy work environments characterized by virtuous organizational practices may be associated with nurses’ willingness to engage in proactive workplace behavior.

The present findings demonstrated a significant positive association between organizational virtuousness and proactive work behavior, supporting H1. One possible explanation is that organizational virtuousness creates a work environment characterized by trust, compassion, integrity, and psychological safety, enabling nurses to express new ideas, anticipate problems, and initiate constructive changes without fear of criticism or negative consequences. Such environments may strengthen employees’ intrinsic motivation and sense of responsibility toward improving patient care and organizational performance. This interpretation is consistent with the JD‐R model, which suggests that positive organizational resources enhance employees’ motivation and discretionary work behaviors. The present findings are consistent with previous studies reporting positive relationships between organizational virtuousness and positive outcomes such as job performance, job satisfaction, well‐being, and organizational commitment [9, 10, 40]. The current study extends these findings by demonstrating that organizational virtuousness is also positively associated with proactive work behavior among nurses. Nevertheless, because of the cross‐sectional design, these findings should be interpreted as associative rather than causal.

The findings also supported H2, demonstrating a significant positive association between organizational virtuousness and psychological well‐being. This relationship may be explained by the fact that virtuous organizations provide supportive interpersonal relationships, ethical leadership, respectful communication, and positive workplace experiences that reduce emotional strain while promoting positive psychological functioning. According to Affective Events Theory, positive workplace experiences shape employees’ emotional responses and psychological well‐being, which may explain why nurses working in virtuous environments reported higher levels of psychological well‐being. These findings are consistent with previous studies showing that organizational virtuousness is positively associated with employee well‐being [10, 25]. While previous research has largely focused on individual determinants such as resilience, stress management, and mindfulness [41, 42], the present findings highlight the importance of organizational factors as potential contributors to nurses’ psychological well‐being.

Consistent with H3, psychological well‐being was positively associated with proactive work behavior. Nurses experiencing higher levels of psychological well‐being may possess greater emotional resilience, cognitive flexibility, and motivational resources, enabling them to recognize opportunities for improvement, solve problems proactively, and adapt effectively to changing clinical demands. From the perspective of the JD‐R model, psychological well‐being may represent a personal resource that facilitates proactive and innovative behaviors. This interpretation is consistent with previous evidence demonstrating positive relationships between psychological well‐being, work performance, and innovative behavior among healthcare professionals [43, 44].

The findings further indicated that organizational virtuousness was significantly associated indirectly with proactive work behavior through psychological well‐being, supporting H4. This suggests that one pathway through which organizational virtuousness may be associated with proactive work behavior is by fostering nurses’ psychological well‐being. From a theoretical perspective, this finding is consistent with the integration of the JD‐R model, Social Exchange Theory, and Affective Events Theory, which collectively propose that supportive organizational resources promote positive psychological functioning, thereby encouraging employees to reciprocate through constructive discretionary behaviors. However, given the cross‐sectional design, this indirect association should be interpreted as a statistically supported, theory‐informed relationship rather than evidence of a causal mediation process. This finding suggests that nurses who perceive greater organizational virtuousness may also report better psychological well‐being, which in turn is associated with higher levels of proactive work behavior. The finding is broadly consistent with previous studies that have highlighted the role of well‐being in explaining relationships between workplace factors and employee outcomes [45, 46].

Importantly, psychological well‐being only partially accounted for the association between organizational virtuousness and proactive work behavior, suggesting that additional mechanisms are likely involved. Organizational virtuousness is a multidimensional organizational resource that may influence employee behavior through several complementary pathways beyond psychological well‐being. For example, nurses working in virtuous organizations may experience greater psychological empowerment, stronger work engagement, higher organizational identification, increased trust in leadership, and more effective knowledge sharing, all of which have previously been associated with proactive workplace behavior [44, 47]. Future longitudinal studies should therefore examine these alternative mediating mechanisms to develop a more comprehensive understanding of how organizational virtuousness promotes proactive work behavior in nursing settings. From a theoretical perspective, the results may be interpreted through the JD‐R model, which proposes that workplace resources are associated with employee well‐being and work‐related outcomes.

### 5.1. Limitations and Future Research

This study has several limitations that should be considered when interpreting the findings. First, because this study employed a cross‐sectional design, the temporal ordering of organizational virtuousness, psychological well‐being, and proactive work behavior could not be established. Consequently, the indirect effects identified in the mediation model should be interpreted as statistical associations based on the proposed theoretical framework rather than evidence of causal mediation. Future longitudinal, multiwave, or experimental studies are required to confirm the temporal sequence and causal mechanisms underlying these relationships.

Second, the study employed a single‐center convenience sampling approach within a specific healthcare institution in Egypt. Although this approach facilitated access to the target population, it may limit the external validity and generalizability of the findings to other healthcare settings, organizational cultures, and nursing populations. Future studies should incorporate multicenter and probability‐based sampling techniques to enhance external validity and enable cross‐institutional comparisons.

Third, data were collected using self‐reported questionnaires, which may introduce response‐related biases, including social desirability bias and common method variance. Although procedural and statistical approaches were applied to reduce and assess common method bias, its potential influence cannot be fully ruled out.

Fourth, the exclusive focus on nurses from a single cultural and organizational context may limit the transferability of the findings to other healthcare systems. Cross‐cultural comparative studies are therefore recommended to examine the robustness of the proposed model across diverse settings.

Finally, the present study focused primarily on proactive work behavior as the main outcome of organizational virtuousness. While this contributes to the existing literature, future research should extend the model by examining additional relevant outcomes such as psychological resilience, employee voice behavior, organizational commitment, and innovative work performance. Such extensions would provide a more comprehensive understanding of the broader organizational and psychological effects of workplace virtuousness in nursing contexts.

### 5.2. Implications of the Study

The present findings suggest that organizational virtuousness is positively associated with nurses’ proactive work behavior and psychological well‐being. Although causal relationships cannot be inferred, the findings may provide useful insights for nursing managers and healthcare leaders seeking to promote positive workplace environments. Healthcare organizations may consider fostering organizational values such as integrity, compassion, trust, and forgiveness through supportive leadership practices, respectful communication, recognition initiatives, and organizational policies that encourage ethical and collaborative workplace behavior. In addition, organizations may consider supporting nurses’ psychological well‐being through initiatives such as stress management, resilience‐building, peer support, or employee assistance programs. However, because these strategies were not evaluated in the present study, their effectiveness should be established through future longitudinal and intervention studies.

## 6. Conclusion

The present study suggests that organizational virtuousness may be associated with nurses’ proactive work behavior and psychological well‐being. These findings indicate that fostering virtuous organizational practices could be considered as one potentially relevant approach to supporting positive work‐related outcomes in healthcare settings. However, given the cross‐sectional design and other methodological limitations, the results should be interpreted as evidence of association rather than causation. Further research using more rigorous designs is needed to confirm these relationships and to determine whether organizational virtuousness contributes to sustained improvements in employee well‐being and organizational functioning.

## Funding

The authors received no financial support for the research, authorship, and/or publication of this article.

## Disclosure

All authors have approved the final version of the manuscript and fulfilled the authorship criteria recommended by the ICMJE, including substantial contributions to the conception and design, acquisition and analysis of data, and critical revision of the article for important intellectual content.

## Conflicts of Interest

The authors declare no conflicts of interest.

## Data Availability

The data that support the findings of this study are available on request from the corresponding author. The data are not publicly available due to privacy or ethical restrictions.
